# Ovarian teratoma in children: a plea for collaborative clinical study

**DOI:** 10.1186/s13048-018-0448-2

**Published:** 2018-08-30

**Authors:** Justyna Łuczak, Maciej Bagłaj

**Affiliations:** Pediatric Surgery and Urology Department, Wroclaw Medical University, 52 M. Sklodowskiej – Curie ST, 50-369 Wroclaw, Poland

**Keywords:** Ovarian teratoma, Ovarian neoplasms, Child

## Abstract

**Background:**

Although teratomas are the most common histologic subtype of childhood ovarian germ cell tumors, their appropriate treatment in this age group still remains unclear. Paucity of research dedicated exclusively to both mature and immature teratomas of the ovary, contribute to decision making difficulties.

Therefore, we decided to review retrospectively our experience in treatment of pediatric ovarian teratomas in order to assess the epidemiology, presenting features, and diagnostic as well as surgical management of these lesions.

**Results:**

The study comprised 58 patients. Fifty percent of patients were between 9 and 15 years old. Mature teratoma was diagnosed in 55(94.83%) patients, while 3(5.17%) patients presented with immature teratoma. Twenty eight (50.91%) girls with mature teratoma had laparotomy and 23 (41.82%) had laparoscopy performed as an initial operative approach. Ovarian tissue sparing technique (preservation of the ovarian tissue of the affected gonad) was applied in only 11.11% of patients operated in the first study period (years 1999–2003) and increased to 40.54% in the second half of our study (years 2004–2016). The extent of gonadal resection was not related with the size of the lesion. Bilateral lesions were noted in 8 patients with mature teratoma. All girls with immature teratoma were subjected to formal laparotomy. Two patients had stage III of the disease and one had stage IV. They underwent at least resection of the affected gonad. Adjuvant chemotherapy was given to all girls with immature teratoma after the surgery.

**Conclusions:**

Under particular conditions ovarian-sparing surgery might be successfully applied in children with mature teratoma. Laparotomy is the treatment of choice in large masses, suspicious for malignancy and if surgical staging is required. High quality prospective multi-institutional studies are required in order to get an objective insight into biology and prognostic factors of teratomas in children.

## Background

Ovarian teratomas are the most common type of ovarian tumors in children. Nevertheless, some aspects of its pathology, classification and management still remain unclear. Their embryology and genetic basis are not yet understood. Specific concerns regard their malignant potential and therefore the possible use of ovarian-sparing operative techniques and the suitability of chemotherapy in its treatment. According to the recent studies, ovarian-sparing procedures are recommended in case of mature teratoma, on specific conditions [1, 2]. However, variety of tissue architecture in teratomas creates a lot of uncertainty. Such as the significance of grade of immaturity or detection of Yolk sac tumor microfoci. In turn, difficulties in histologic sampling might result in overlooking of some malignant cells, especially in the large tumors. Although surgery remains the mainstay of treatment, the indications for chemotherapy vary between the studies [2–7]. Paucity of research dedicated exclusively to both mature and immature teratomas of the ovary, contribute to decision making difficulties. Therefore, we decided to review our experience of pediatric ovarian teratomas at our institution in order to assess the epidemiology, presenting features, and diagnostic as well as surgical management of these lesions.

## Material and methods

We analyzed retrospectively medical files of all consecutive patients aged 0–18 years, who underwent surgical procedures for ovarian teratoma between 1991 and 2016 at the University Department of Pediatric Surgery and Urology in Wroclaw, Poland. The demographic data, presenting symptoms and signs, results of laboratory and diagnostic studies (including ultrasound examination, additional imaging studies and tumor markers), details of surgical procedures, and clinical outcomes (including preservation rate), were extracted in each case. Ovarian mass characteristics were evaluated by preoperative imaging (structure and size) or by description of the procedure (size). An ovarian lesion was described arbitrarily as large when its diameter was 10 cm or more in girls aged between 1 and 18 years and 5 cm or more in newborns and infants. Such classification was based on the previous experience of other authors, in order to obtain comparable results [8, 9]. A choice of an operative technique (either laparoscopic or open) depended solely on a surgeon’s preference. The extent of gonadal resection was based on intraoperative findings and it ranged from total, when the whole gonad affected by lesion was removed, to partial resection, when at least the remnant of ovarian tissue was preserved. Preservation rates were compared taking into consideration the operative method, the histological type, the size of the mass, the presence of ovarian torsion and the study period (1991–2003 vs 2004–2016). The analyzed data were subjected to the statistical evaluation with Chi square, Kruskal-Wallis and Barttlet test, Pearson’s correlation coefficient and logistic regression. The statistical software package EPIINFO Ver. 7.1.1.14 was used for all data analyses.

## Results

### Epidemiology and presentation

**Fig. 1:**
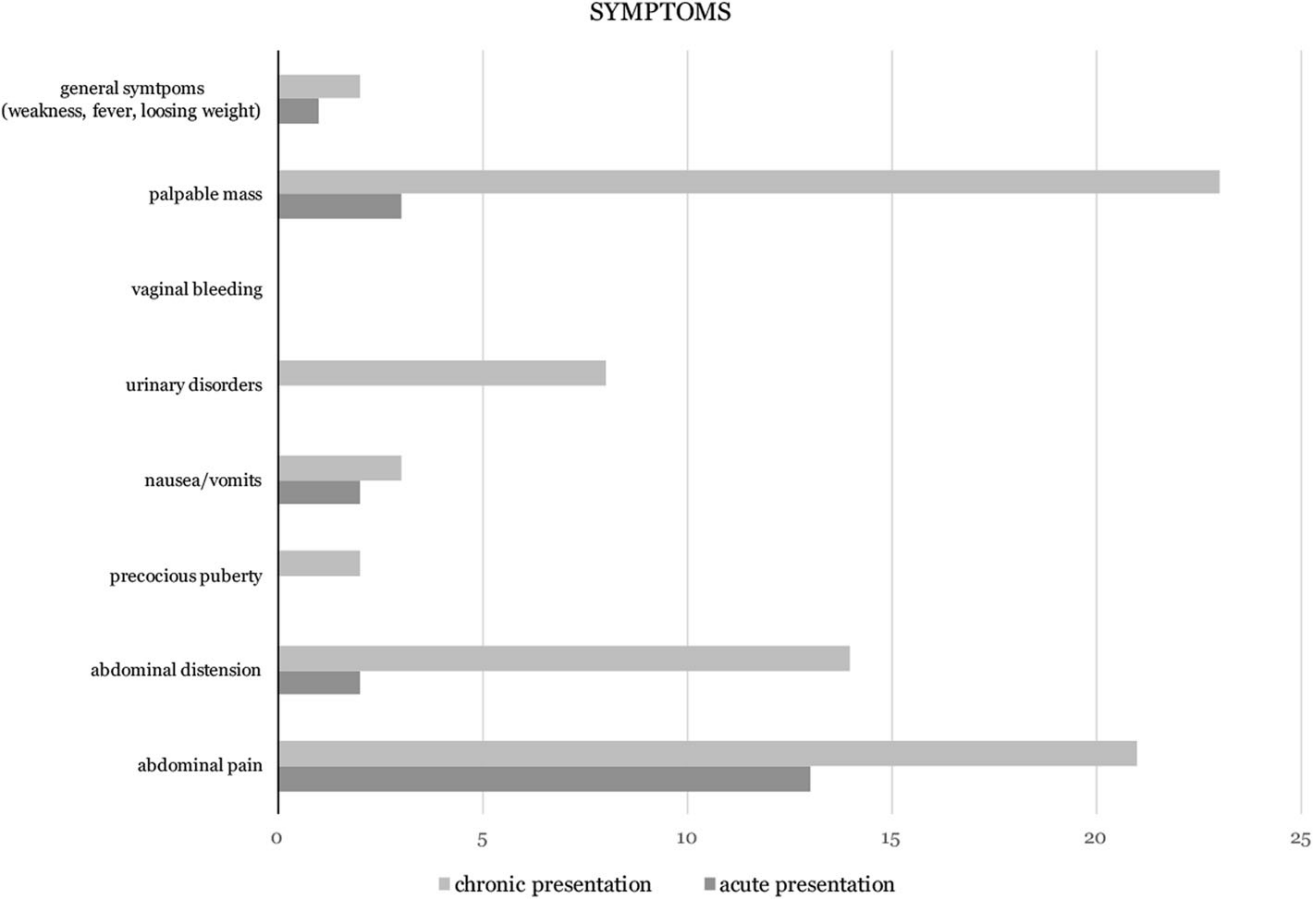
The most common presented symptoms

The study comprised 58 patients. The median of age was 12,0 years (SD = 4.2). Fifty percent of patients were between 9 and 15 years old. Mature teratoma was diagnosed in 55(94.83%) patients, while 3(5.17%) patients presented with immature teratoma (age: 8,12 and 15 years old). There were 44 girls (75.86%) with chronic presentation (no symptoms requiring immediate surgical intervention). Fourteen patients (24.14%) presented with acute symptoms (admitted to the hospital as emergency due to relevant pain or/and vomits, fever). Abdominal pain, palpable mass and distension were the most frequent clinical features noted in the whole study group. Palpable mass was the most frequent symptom in girls with chronic presentation (52.27%), while abdominal pain predominated in girls with acute presentation and was noted in 13 of them (92.86%); Fig. 1. The most common intraoperative finding additional to the tumor, in girls with acute presentation, was ovarian torsion (85.71% of patients with acute symptoms; *p* = 0.00634, χ^2^ = 7.45, Rr = 3.83, CI = 1.67÷8.82); Table 1. All three patients with immature teratoma presented with chronic symptoms.

**Table 1:** Comparison of the clinical data between mature and immature teratomas

Overall			
Number of patients	58		
Age(years)	12.0 (9.0÷15.0); sd 4.2		
	Mature teratoma	Immature teratoma	
Number of patients	55	3	
Symptoms			
Acute presentation (number of patients)	14(24.14%)		
Abdominal pain	13		
Palpable mass	3		
Distension	2		
Other	1		
Chronic presentation (number of patients)	44(75.86%)		
Abdominal pain	21		
Palpable mass	23		
Distension	14		
Other	15		
US result			
Solid	2	1	
Complex	38	1	
Cystic	14	0	
Size of the lesion			
Large lesion	28	3	
Lesion that was not described as large	16	0	
Tumor markers			
Positive	5	3	
AFP	1	3	
CA-125	4	0	
Negative	33	0	
Bilateral lesion			
Synchronous	4	0	
Metachronous	2	0	
Ovarian torsion	Mature teratoma	Immature teratoma	*p* value
Acute presentation	7(85.71%)	0	0.00634
Chronic presentation	5(11.36%)	0	

### Diagnostic studies

#### Mature teratomas

**Fig. 2:**
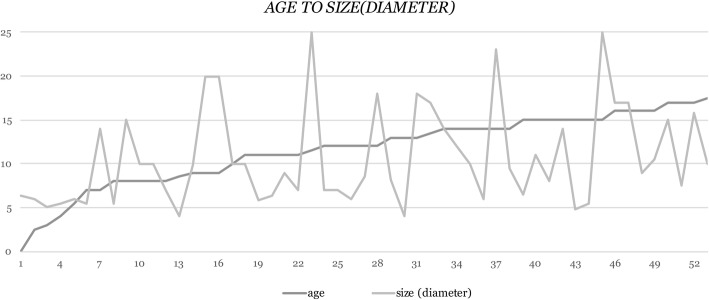
Patient’s age and size of the lesion

All of the patients underwent abdominal ultrasound scan (US). It showed a cystic structure in 14 girls (25.45%). A complex ovarian mass was noted in 38 (69.09%) and a solid mass in 2 girls (36.36%). Twenty-eight girls (50.91%) had a large tumor (with a diameter of more than 10 cm). Twenty-one patients (38.18%) had computed tomography (CT) or magnetic resonance(MRI) studies performed preoperatively. In 11 patients this additional examination led to the evaluation of tumor origin or its real structure changing the initial radiological description of the lesion (based on US scan). Statistical analysis revealed linear dependence between the size of the lesion and patient’s age (Pearson product-moment correlation coefficient: *r* = 0.29, *N* = 53, *p* = 0.039, Fig. 2). The results of tumor markers evaluation (AFP – alpha-fetaprotein, β-hCG - beta subunit of human chorionic gonadotropin, CA125 - cancer antigen 125, LDH - lactate dehydrogenase) were available for 38 girls (69.09%). They were elevated in 5 girls (9.09%). Only one marker was positive in four patients out of five: CA-125. We noted elevated level of AFP (37.45 ng/ml) in one patient aged 14 (Table 1).

#### Immature teratomas

Abdominal US showed a complex ovarian lesion and a solid mass in one girl each. A heterogenous structure of one of the lesions was confirmed in CT. One patient had only computed tomography result available, it revealed a complex lesion. Calcifications were observed on imaging in two patients. Large tumor was diagnosed in all three girls. Tumor markers evaluation revealed elevated level of AFP in all girls (186,1680 and 6434 ng/ml).

### Treatment

#### Mature teratomas

Twenty-eight (50.91%) girls had laparotomy and 23 (41.82%) had laparoscopy performed as an initial operative approach. Conversion to open procedure was noted in 4 girls (7.27%). The number of patients operated on with laparoscopy increased from 44.44 to 51.35% in the second part of our study (years 1991–2003 vs 2004–2016). Ovarian tissue sparing technique (preservation of the ovarian tissue of the affected gonad) was applied in only 11.11% of patients operated in the first study period and increased to 40.54% in the second half of our study. The preservation rate was higher in the group of girls in whom laparoscopic technique was chosen. The extent of gonadal resection was not related with the size of the lesion. Moreover, tissue sparing was possible in 32.26% of those with large lesions and 29.17% of girls with lesions not classified as large. The difference was not statistically significant; *p* = 0.806, χ2 = 0.605 (Table 2). Biopsy procedure was performed in 21 cases and it was negative in all of them.

**Table 2:** Ovarian tissue preservation rates in selected groups of patients

Study period	Overall	Operation method		Large lesion		Torsion	
		Laparoscopy	Laparotomy	*–*	*+*	*–*	*+*
1991–2016	29.31%	43.48% (27.27% large lesions)	20.00% (75.76% large lesions)	29.17%	32.26%	30.43%	25.00%
				*p* = 0.806			
1991–2003	11.11%	14.28%	7.69%	12.50%	9.09%	7.14%	0.00%
2004–2016	40.54%	56.25%	27.27%	37.50%	45.00%	37.50%	50.00%

#### Immature teratomas

All girls with immature teratoma were subjected to formal laparotomy. Two patients had stage III of the disease and one had stage IV (according to Children’s Oncology Group ovarian staging system) [10]. They underwent at least resection of the affected gonad. Biopsy procedure was performed all girls and it was negative in all of them.

### Histopathologic findings

#### Mature teratomas

In 18 cases there was ovarian dermoid cyst diagnosed.

#### Immature teratomas

Grading (according to O’Connor and Norris) was available only in one patient (G1) [11]. No Heifetz lesions were observed.

### Final outcome

#### Mature teratomas

Bilateral, synchronous lesions were noted in 6 patients with mature teratoma. Metachronous disease in the contralateral ovary was observed in two patients with mature teratoma after the first operation within 10 months and 5 years. Respectively (Table 1). Follow-up was possible in 13 patients and lasted between 1 and 12 years. All girls which have at least one ovary have normal menstruation. Some of them (30.77%) reported painful menstruations or are diagnosed with simple cysts (without operative treatment).

#### Immature teratomas

Adjuvant chemotherapy (protocol TGM 95) was given to all girls with immature teratoma after the surgery. All three patients responded well to the treatment and were disease free after follow-up of 3,4 and 12 years respectively.

## Discussion

Teratomas present diverse biological behavior and continue to be the cause of many diagnostically and therapeutically challenging issues. While it is commonly accepted that mature teratomas of the ovary are benign neoplasms, the classification of the immature ones is still discussed. About 25% of all the pediatric GCTs present as tumors with more than one histologic type. In this situation, therapy and prognosis depend on the component with the highest malignancy [12–17]. In accordance with this approach pure immature teratomas were excluded in the recent studies concerning pediatric malignant germ cell tumors. Conversely, patients with malignant germ cell tumors, even when these lesions contained mature teratoma or immature teratoma elements, were not eligible for studies concerning teratomas exclusively [2, 12, 17, 18]. Nevertheless, there are also studies were a lesion was considered as immature teratoma although the pathology report revealed a component of another malignant tumor [19]. Marks of the controversy regarding this topic are also reflected in the nomenclature of these lesions across the studies. The multiplicity of names describing immature teratomas (immature teratoma, malignant teratoma, teratoma with malignant elements, immature teratoma with malignant behavior) renders universally applicable classification of these lesions very difficult [5, 12, 17–21].

All the girls in our study had at least stage III of the disease. In a study from 2012, Schneider et al. ascertain that immature teratomas behave in a malignant fashion only if foci of malignant germ cell elements are present and if they are resected incompletely. According to their paper, tumors containing clusters of yolk sac tumor are likely responsible for the reports that immature teratoma may metastasize [2]. Nevertheless, in some recently published studies immature teratomas are still included in the evaluation of patients with malignant germ cell tumors and even at US National Cancer Institute website reads that “Immature teratomas can exhibit malignant behavior and metastasize” [7, 14, 22, 23]. Therefore, can we definitively classify them as benign?

Another important aspect in attempts to estimate the real nature of these lesions is the importance of the grade of immaturity. In a study published by Malignant Germ Cell Tumor International Collaborative it was revealed that grade was the most important risk factor for relapse in ovarian immature teratoma [6]. However, there is scarceness of papers reporting the use of a detailed grading system and its significance in children has been challenged by some former studies. Additionally, high immaturity itself is not associated with a poor prognosis if the tumor is completely resected [2]. Defining real nature of the lesion is indispensable in choosing optimum management. Obtaining the correct pathology report is crucial here. Clusters of malignant tumor can be easily overlooked and yolk sac tumor components might be very small. Microfoci of yolk sac tumor (Heifetz lesions) are not precisely defined among the studies, thereby classifying a lesion as malignant yolk sac tumor is particularly difficult [2, 5, 12]. Performing a central pathomorphological examination can be helpful in the case of these neoplasms. Perhaps new tumor markers, like the ones discovered by Feng et al. will help eliminate mistakes in pathomorphological diagnoses [24]. Grading was available only in one patient in our study and no Heifetz lesions were observed. This is a retrospective study and we cannot verify the results of the pathology report. Taking into consideration that all immature teratoma cases were of stage III at least, it is questionable if no other malignant components were present.

Despite these unclear pathological aspects, commonly applied diagnostic work-up of ovarian teratomas include physical examination, ultrasound imaging and tumor markers evaluation. In most of the studies the peak incidence of these lesions is reported in early adolescence [2, 12]. It was also confirmed by our study. Symptoms of ovarian teratoma do not differ from those observed in other ovarian masses. Some patients reveal acute symptoms rising suspicion of ovarian torsion. The risk of torsion complicating a case of ovarian teratoma is approximately 3 to 16% in children [2, 25]. Those cases are rarely associated with malignancy. Tsai et al. revealed that young girls tend to have either torsion of a mature cystic teratoma or torsion without underlying condition, while older patients are more likely to present with torsion and a tumor [2, 26]. In our study ovarian torsion was the most common intraoperative finding additional to the tumor in girls with acute presentation. The incidence of ovarian torsion was not higher in older girls (Kruskal-Wallis test, *p* = 0,765).

As most of the mature teratomas are slow-growing cystic lesions and there are some common characteristic findings, they are easily recognized on US. Nevertheless, their typical features might be less clear in case of prepubertal girls and when the lesion is large [13, 26, 27]. Our study poses limitations in that respect. The ultrasound description was often very short and we were unable to identify characteristic features for teratoma in each case. Diagnosis of immature type also poses problems. The appearance of the tumor on ultrasound is non-specific, although predominance of solid component is a differentiating factor. Identification of fat, mural nodules and calcified components within the lesion is typical for teratomas. However, there are reports suggesting that typical CT features of teratomas occur more often in mature than in immature lesions. Those findings suggest that CT or MRI imaging might be helpful, rather for staging and assessing of tumor respectability [13, 28, 29].

Alphafetoprotein evaluation is an important prognostic factor in many malignant germ cell tumors treatment protocols and it is also used in follow-up of those patients. Additionally, it is a characteristic marker of yolk sac tumor. Regarding its levels in ovarian teratomas, it was revealed that they are rarely elevated [2, 12]. In the recent Malignant Germ Cell Tumor International Collaborative study regarding ovarian immature teratomas, patients with AFP levels higher than 1000 ng/mL were excluded because this level was considered more likely to indicate malignant elements [6]. However, this approach was questioned by Terenziani et al. who used an AFP level cutoff “high for age” in their study [14]. Another limitation is caused by the vide variation in AFP levels at birth and the variability in its half-time within the first year of life [2]. Multiple publications where cases of immature teratoma are analyzed without taking AFP levels into consideration makes it more difficult to form a credible assessment of this indicator [14, 30, 31]. Only one girl in the immature teratoma group had AFP levels < 1000 ng/mL in our study. Given the results of the recent research this is another factor calling into question the result of pathology report in our cases.

According to the experience of many pediatric surgical centers, surgery remains the mainstay of treatment in ovarian teratomas. Under particular conditions ovarian-sparing surgery might be successfully applied. Preservation of ovarian tissue should be reserved for cases of localized mature teratoma, when there is a plane of dissection between the tumor and the normal ovary [1, 2, 13, 14, 32]. The study by O’Neill et al. revealed that normal ovarian tissue was visualized on follow-up ultrasound after cystectomy even if there was no normal ovarian tissue visible preoperatively [13]. Although incomplete resection is an important risk factor of recurrence, relapse is not inevitable in those cases. The use of laparoscopic techniques in the treatment of ovarian teratomas has its supporters and opponents. If the suspicion of malignancy is low and a surgeon is experienced in minimal invasive surgery, laparoscopic approach might be adequate [2, 6, 7, 12, 15, 21, 33]. Children’s Cancer Study Group demonstrated in their study that cyst fluid aspiration or spillage during surgery are not associated with relapse, thereby this factor, claimed by laparoscopy opponents as more often associated with minimal invasive methods, might not have such importance for the final outcome [12, 30]. Our results are promising in that respect. We revealed that the use of laparoscopy increased in time and the preservation rate was higher in the laparoscopy group. Laparotomy is the treatment of choice in large masses, suspicious for malignancy, if surgical staging is required [13]. The preservation rate seemed no to be affected by the size of the lesion in our study.

An important aspect of the management of ovarian teratomas is their recurrence rate and the incidence of bilateral lesions. The recurrence rate after cystectomy vary between 3 to 13%. However, in the study were the recurrence rate was estimated to be of 10% the authors revealed that only 3% of the recurrent cases will require reoperation [13, 34]. There were studies suggesting that women with bilateral or multiple dermoid cysts may include a subgroup of patients with a greater tendency to develop future ovarian germ cell neoplasms. Due to low risk of malignant transformation in case of mature cystic teratomas in children the treatment should be directed on the basis of age, fertility desire or presence of another pelvic pathology rather than the size or bilaterality [35–37]. In the study by O’Neill et al. the biopsy of the contralateral macroscopically normal ovary revealed pathological lesions only in 1.1% of patients. Therefore, preoperative ultrasound in combination with careful inspection of the contralateral ovary at the time of surgery offer a safe alternative to biopsy [13, 38]. Our results support this approach, none of the biopsies was positive in our study. Given the sensitivity of ultrasound in the detection of mature cystic teratomas, annual imaging seems appropriate as a postoperative surveillance. AFP monitoring is not recommended in case of completely resected ovarian teratomas without preoperatively elevated tumor markers [12, 38].

The treatment of immature teratomas poses much more difficulties. Their possible malignant behavior requires appropriate risk classification. Among factors considered as those influencing the prognosis, except the previously mentioned, staging is the next unclear one. Since malignant tumors and benign ones cannot be distinguished based only on intraoperative appearance, there are studies recommending staging in all tumors [2, 39]. However, Billimire et al. revealed that survival appears to have been unaffected by deviations from surgical guidelines when chemotherapy is administrated [39]. Different staging systems across the surgical centers renders evaluation of their significance difficult [2, 6]. Another controversial aspect is the use of chemotherapy. Number of therapeutic options might be found in the literature. To name some of them: chemotherapy for recurrence, only for malignant recurrence, in all cases of stage I grade 2 and higher or only in girls older than 15 [2, 6, 7, 22, 40]. In the German MAKEI study more than half of the children with tumor recurrence after watch and wait strategy had yolk sac tumor in addition to teratoma. It might be one of the main counter-arguments to the results of the studies which did not find evidence that chemotherapy had any significant therapeutic effects in pure teratomas and that it does not decrease relapses in pediatric population [40]. Recently created Malignant Germ Cell Tumor International Collaborative in its publications gathering data from four clinical trials recommends surgery alone for patients with stage I/II, grade 1/2 tumors and suggests conducting of a prospective trial of observation after surgery for patients with grade 2/3, stage II-IV tumors [6]. This approach might be encouraged by the results of Park et al. as they revealed that most stage I malignant ovarian germ cell tumor recurrences can be successfully salvaged by surgery and BEP chemotherapy without compromising the overall survival [22]. Adjuvant chemotherapy according to protocol TGM 95 was given to all girls with immature teratoma in our study group. They all responded well to the therapy but it remains questionable if these were real pure immature teratoma lesions.

Schneider et al. revealed that patients with cytogenetically abnormal immature teratomas often develop recurrence [2]. An optimal consensus treatment strategy should be based on a detailed analysis of all possible factors. In this regard, the above mentioned study shows that we might be still not aware of a number of them. The future molecular research may allow distinguishing low-risk from high-risk patients accurately [15].

Different staging systems, histopathologic classification systems and risk stratification are important factors impeding development of an appropriate treatment protocol [2, 12]. Additional factors include the lack of consistent terminology in the literature and analyzing pediatric and adult patients together [5, 20, 31]. International cooperation seems the only way to progress in the prospective knowledge of prognostic factors for these tumors. It will not be possible without high quality and meticulous diagnostic approach in all participating institutions.

## Conclusions

Under particular conditions ovarian-sparing surgery might be successfully applied in children with mature teratoma. Laparotomy is the treatment of choice in large masses, suspicious for malignancy and if surgical staging is required. High quality prospective multi-institutional studies are required in order to get an objective insight into biology and prognostic factors of teratomas in children.
